# Reactivation capacity of TiO2 nanoparticles for dental bleaching: an *in vitro* study

**DOI:** 10.1590/0103-644020266525

**Published:** 2026-08-07

**Authors:** Suely Cristina Aragão Veras dos Santos, Tainah Oliveira Rifane, Isabel Silva do Nascimento, Lucca Reis Mesquita, Nayara Oliveira de Souza, Madiana Magalhães Moreira, Victor Pinheiro Feitosa

**Affiliations:** 1 Paulo Picanço School of Dentistry, Fortaleza, Ceará, Brazil; 2 Department of Operative Dentistry, University of Iowa, College of Dentistry, Iowa City, IA, USA

**Keywords:** tooth bleaching agents: hydrogen peroxide, titanium

## Abstract

This study evaluated the bleaching efficacy, pH behavior, and reuse potential of a titanium dioxide nanoparticle (TiO₂) suspension as a photocatalytic, peroxide-free alternative to carbamide peroxide (CP) for at-home bleaching. Forty bovine incisors were subjected to a 21-day at-home bleaching regimen (2 h/day) using fresh or reused agents (n = 10): fresh CP (16% CP), reapplied CP (16% CP collected and reused), fresh TiO₂ (10 wt% TiO₂ suspension), and reapplied TiO₂ (reactivated 10 wt% TiO₂ suspension). TiO₂ suspensions were UV-activated daily (385 nm, 50 min), and the reused TiO₂ was reactivated prior to each application. The pH of the bleaching agents was assessed before gel application and after completion of the at-home protocol; all experimental groups showed a similar pH trend, with values increasing from 6.0 to 7.0 throughout the procedure. Bleaching efficacy was evaluated using CIEDE2000 (ΔE₀₀) and whiteness index (ΔWID) values obtained with a digital spectrophotometer (VITA Easyshade V). Data were analyzed with one-way ANOVA and Tukey’s test (α = 0.05). ΔE₀₀ analysis showed that fresh TiO₂ produced the lowest color change, with values closest to the perceptibility and acceptability thresholds, differing significantly from reapplied TiO₂, fresh CP, and reapplied CP (p < 0.05). No significant differences were observed among the remaining groups, and all other conditions exhibited ΔE₀₀ values above the acceptability threshold. ΔWID values did not differ significantly among groups (p = 0.475). The UV-activated TiO₂ suspension (100 min total exposure) was able to bleach teeth without peroxide.

## Resumo

Este estudo avaliou a eficácia clareadora, o comportamento de pH e o potencial de reaplicação de uma suspensão de nanopartículas de dióxido de titânio (TiO₂) como alternativa fotocatalítica e isenta de peróxido ao peróxido de carbamida (CP) para clareamento dental caseiro. Quarenta incisivos bovinos foram submetidos a um regime de clareamento caseiro de 21 dias (2 h/dia) utilizando agentes frescos ou reutilizados (n = 10): CP fresco (16% CP), CP reaplicado (16% CP coletado e reutilizado), TiO₂ fresco (suspensão de TiO₂ a 10% em peso) e TiO₂ reaplicado (suspensão de TiO₂ a 10% reativada). As suspensões de TiO₂ foram ativadas diariamente por luz UV (385 nm, 50 min), e o TiO₂ reutilizado foi reativado antes de cada aplicação. Os níveis de pH dos agentes clareadores foram avaliados antes da aplicação do gel e após a conclusão do protocolo caseiro, e todos os grupos experimentais apresentaram tendência semelhante, com aumento de pH de 6,0 para 7,0 ao longo do procedimento. A eficácia do clareamento foi avaliada por meio dos valores CIEDE2000 (ΔE₀₀) e do índice de brancura (ΔWID), obtidos com um espectrofotômetro digital (VITA Easyshade V). Os dados foram analisados por ANOVA de uma via e teste de Tukey (α = 0.05). A análise de ΔE₀₀ mostrou que o TiO₂ fresco produziu a menor alteração de cor, com valores mais próximos aos limiares de perceptibilidade e aceitabilidade, diferindo significativamente do TiO₂ reaplicado, CP fresco e CP reaplicado (p < 0.05). Não foram observadas diferenças significativas entre os demais grupos, e todas as outras condições apresentaram valores de ΔE₀₀ acima do limiar de aceitabilidade. Os valores de ΔWID não diferiram significativamente entre os grupos (p = 0.475). A suspensão de TiO₂ ativada por UV (100 min de exposição total) demonstrou capacidade de promover clareamento dental sem o uso de peróxido.

**Figure:**
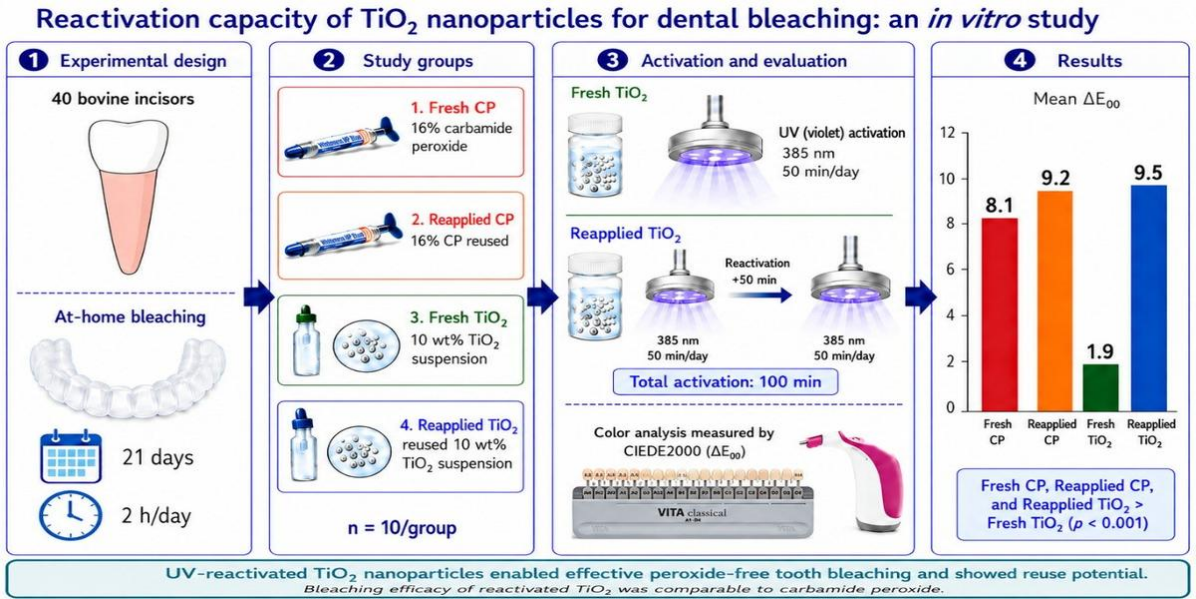

## Introduction

Dental bleaching (DB) is a minimally invasive procedure within aesthetic dentistry ^1^ ^,^ ^2^ ^,^ ^3^. Different techniques include in-office, at-home, and combined approaches, all employing bleaching agents that oxidize organic pigments ^4^ ^,^ ^5^. The most widely used agents are hydrogen peroxide (HP) and carbamide peroxide (CP), which are applied at varying concentrations depending on the technique ^6^. CP decomposes into HP and urea, with HP acting as the primary active compound in most DB products ^7^ ^,^ ^8^ ^,^ ^9^. The proposed mechanism involves HP diffusion into dental tissues, where free radicals break down pigments into smaller, more soluble molecules, resulting in a color change ^7^ ^,^ ^8^ ^,^ ^9^. An alternative hypothesis suggests that bleaching may also be related to modifications in dentin organic matrix polypeptides ^1^ ^,^ ^2^ ^,^ ^3^.

Considering the characteristics of bleaching agents, a systematic review has demonstrated that a carbamide peroxide (CP) formulation reduces the risk of sensitivity while still achieving satisfactory color changes ^5^ ^,^ ^10^. However, one of the primary and most common side effects of dental bleaching is post-operative sensitivity and increased pulpal inflammation, which are frequently observed in certain patients due to high HP concentrations ^11^ ^,^ ^12^ ^,^ ^13^. Additionally, DB with HP may compromise enamel surface roughness, microhardness, and fracture resistance ^1^ ^,^ ^4^ ^,^ ^5^. Consequently, current approaches aim to mitigate this issue by either reducing HP concentration through its association with particles such as niobium, zinc dioxide, chitosan, titanium dioxide (TiO_2_), and nitrogen, or by developing whitening materials devoid of peroxides altogether ^14^ ^,^ ^15^ ^,^ ^16^ ^,^ ^17^.

TiO_2_ has emerged as a promising photocatalytic compound for DB. This semiconductor is non-toxic and can generate free radicals via oxidation when exposed to ultraviolet (UV) light with wavelengths of 385-440 nm ^14^ ^,^ ^15^. Previous studies have shown that incorporating TiO_2_ into visible-light-activated HP gels enhances bleaching speed and results in noticeable color changes ^16^ ^,^ ^17^. Furthermore, experimental bleaching gels containing TiO_2_ have produced satisfactory results in altering tooth coloration and have not exhibited any adverse effects on tooth enamel properties ^14^ ^,^ ^18^. However, there is currently a lack of research investigating the potential of isolated TiO2 as a bleaching agent, without being combined with HP or CP. In a previous study by Rifane TO et al. (2025) ^16^, TiO_2_ nanoparticle suspensions were proposed as a viable peroxide-free alternative for dental bleaching, particularly at higher concentrations and longer photocatalytic exposure times. The TiO_2_ suspension was evaluated in water without peroxide and showed promising bleaching results. In a randomized clinical trial published by Priscila Melo et al.,2025 ^19^, low-concentration bleaching gels containing hyaluronic acid and NF-TiO₂ nanoparticles, activated by violet LED, showed whitening efficacy comparable to a 35% HP gel, with significantly reduced tooth sensitivity and no adverse effects on pulp oxygen saturation.

In this study, the carbamide peroxide gel was reapplied after collection to extend contact time with the dental substrate and ensure effective hydrogen peroxide release. Given hydrogen peroxide's limited half-life, it may still exert a bleaching effect even after the initial application (1-4). Moreover, this strategy enabled methodological standardization within the TiO₂ group, in which the same solution was reactivated with a violet LED.

This prompted further research to determine the optimal concentration of TiO_2_ nanoparticles and to assess whether multiple activation cycles would enhance their effectiveness. Therefore, this research aimed to evaluate the bleaching efficacy and reuse potential of a suspension of titanium dioxide nanoparticles (TiO_2_) as an active photocatalytic agent in an alternative to carbamide peroxide (CP) in at-home bleaching. The study hypothesizes that the bleaching protocol with TiO_2_ ^1^ achieves color changes similar to those of CP 16%, and ^2^ can reuse the suspension after reactivation with UV light. 

## Materials and methods

### Sample preparation

Forty permanent bovine incisors were collected from a local slaughterhouse, cleaned with a periodontal curette, and stored in 0.1% (w/v) thymol solution. The selected teeth were cut on a precision metallographic cutter (Odeme, Luzerna, Santa Catarina, Brazil) to remove the roots. Once cut, wax (nº 7, Lysanda, São Paulo, Brazil) was inserted into the root canals to fill and seal the pulp chamber. After that, the samples were submerged in black tea (Dr Oetker, São Paulo, Brazil) for 1 week at 37°C in an oven. The solutions were prepared in the proportion of 250 mL of water and 18 g of black tea powder, and changed every day. After prophylaxis, the specimens were evaluated for staining homogeneity to standardize and randomly distributed into groups (n=10). The inclusion criteria comprised teeth with A3, B3, and A3.5 colors, as measured by a digital spectrophotometer (EasyShade, VitaZahnfabrik, Bad Säckingen, Germany) ^14^. 

### TiO_2_ suspension preparation

The suspensions were prepared using 30 nm TiO_2_ nanoparticles (predominantly anatase and rutile, 50:50) (Sigma-Aldrich, St. Louis, EUA) at a concentration of 10 wt % in distilled water. The components were accurately weighed using an analytical balance (Bel Engineering®, Milano, Italy). Subsequently, the suspension was stirred in a vortex (LabGenius, China) for 30 seconds to ensure homogeneity between the distilled water and the nanoparticles. Finally, the mixture was stored in a light-protected environment to prevent spontaneous reactions. Before application to the teeth, the suspension underwent activation with ultraviolet (UV) light at 385 nm for 50 minutes at 2 W for photocatalysis (2CPS, Opus, São Paulo, Brazil).

### Division of groups and bleaching protocols

The samples were exposed to at-home bleaching using different fresh or reapplied bleaching agents (n=10): fresh CP - 16% CP; reapplied CP - 16% CP collected with a spatula and reused in other sample; fresh TiO_2_ - 10 % TiO_2_ suspension was subjected to a 50 minutes activation period for photocatalysis prior to being applied to the teeth; or reapplied TiO_2_ - reactivated 10 % TiO_2_ suspension subjected to a 50 minutes reactivation period (totaling 100 minutes) of photocatalysis before being applied to the teeth.

**Figure 1:**
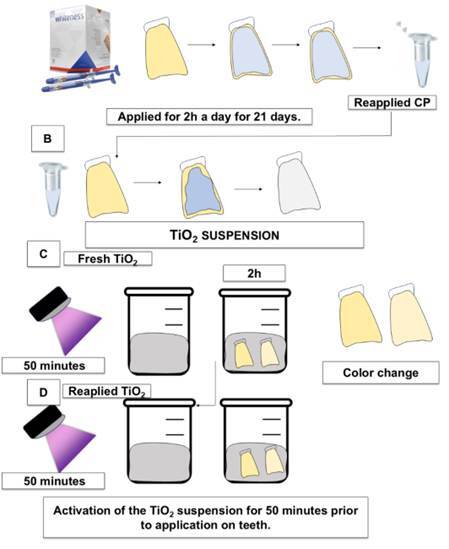
Illustrative diagram of the bleaching protocols. A) fresh CP: the 16% CP gel is applied to the buccal surface of the teeth. B) reapplied CP: 16% CP gel collected after tooth bleaching is stored for reuse in another sample. C) fresh TiO_2_: the suspension underwent photocatalysis for 50 minutes before being applied to the teeth. D) reapplied TiO_2_: the same suspension underwent a second round of photocatalysis for 50 minutes before being applied to different teeth for bleaching, resulting in two activations for this group totaling 100 minutes.

The schematic diagram of the at-home whitening protocol, consisting of 2 hours daily over 21 consecutive days for all groups, is depicted in Figure 1. In the fresh CP group, the 16% CP was applied to the buccal surfaces of the teeth. Following the 2-hour application period, all the bleaching gel on the tooth surfaces was collected with a spatula and stored in Eppendorf tubes. This gel was reapplied to another sample belonging to the reapplied CP group. For the fresh TiO_2_ group, the suspension was subjected to photocatalysis for 50 minutes before being applied to the teeth. The photocatalysis process involved exposing the suspension to ultraviolet light (385 nm wavelength) with an exitance power of 2W and a final irradiance of 200 mW/cm^2^ (2 CPS, Opus, São Paulo, Brazil). The same suspension was subjected to a second photocatalysis session for 50 minutes before being applied to the teeth in the reapplied TiO_2_ group, for a total of 100 minutes across two activations. 

### Color Change Assessment

Color change was assessed at two time points: initially, after staining with black tea and subsequent prophylaxis, and immediately after bleaching. Measurements were performed on the enamel using a digital spectrophotometer (EasyShade, Vita Zahnfabrik, Bad Säckingen, Germany) in the central zone of the crown inside a controlled light box (D65, GTI Graphic Technology, Newburgh, NY, USA). The hue was determined according to the EasyShade parameters, where L* ranges from 0 (black) to 100 (white), and a* and b* represent chromaticity, with a* corresponding to the red-green axis and b* to the yellow-blue axis. Color change was evaluated using CIEDE2000 (ΔE₀₀) and the whiteness index (ΔWID), as shown in the formula below ^20^. For interpretation, ΔE₀₀ values were compared with established perceptibility (ΔE₀₀ = 0.8) and acceptability (ΔE₀₀ = 1.8) thresholds, which indicate the most minor color differences that can be perceived or considered clinically acceptable, respectively.

$${\Delta E}_{00}\;(T_{14}-\;T_{0})\;=\;{\left[{\left(\Delta L'/KLSL\right)}^{2}+{\left(\Delta C'/\;KCSC\right)}^{2}+{\left(\Delta H'/KHSH\right)}^{2}+RT*(\Delta C'/KCSC)*(\Delta H'/KHSH)\right]}^{1/2}$$

WID = 0.511 L* - 2.324 a* - 1.100 b*

In the CIEDE2000 formula, ΔL′, ΔC′, and ΔH′ represent differences in lightness, chroma, and hue, respectively. The weighting functions S 𝐿 , 𝑆 𝐶 , and 𝑆 𝐻 adjust these differences according to human perceptual sensitivity, while the parametric factors 𝑘 𝐿 , 𝑘 𝐶 , and 𝑘 𝐻 (set to 1) standardize the evaluation conditions. The rotation term 𝑅 𝑇 corrects for interactions between chroma and hue, improving the perceptual accuracy of color differences.

The submitted data were subjected to normality and homoscedasticity tests (Shapiro-Wilk and Levene tests). Subsequently, a two-way ANOVA analysis was performed, considering the factors "bleaching agent - CP or TiO_2_ “and "fresh or reapplied mode". Post-hoc Tukey's tests were conducted to examine significant differences further. The statistical analyses were carried out using SigmaStat version 3.2 software, with a predetermined significance level of 5%.

### Quantification of pH in Contact With Enamel Surface

The pH levels of the bleaching agents were evaluated using pH indicator strips (Mquant, Merck, Darmstadt, Germany). Measurements were taken before gel application to the tooth surface and after completing the at-home bleaching protocol. The initial and final pH values correspond to the mean of the readings obtained across the three applications of each bleaching agent. The second measurement was obtained after the 2-hour at-home bleaching procedure concluded.

## Results

Analysis of ΔE₀₀ demonstrated that the 50% Fresh group exhibited significantly lower color change than all other conditions, with values closer to the established perceptibility (~0.8) and acceptability (~1.8) thresholds. Pairwise comparisons showed significant differences between 50% Fresh and 50% Reapplied (p = 0.002), PC16% Fresh (p = 0.011), and PC16% Reapplied (p = 0.008). No significant differences were observed among the remaining groups (p ≥ 0.810), which presented ΔE₀₀ values well above the acceptability limit and were therefore readily perceptible clinically. The WID results are presented in Table 1, and no significant differences were observed among the groups (p = 0.475).

**Table 1:** Means (standard deviations) of color variation expressed in ΔWID.

Groups	PC16%	50%Tio_2_
Fresh	4.30 (0.59) A	3.50 (0.54) A
Reapplied	5.57 (0.67) A	4.48 (0.74) A

Different uppercase letters indicate statistically significant differences among groups within each parameter (p < 0.05).

The pH values obtained before and after the bleaching procedure are presented in Table 2. All groups showed an initial pH of 6.0, which increased to 7.0 following bleaching, with no observable differences among the experimental conditions.

**Table 2:** pH values measured before and after the bleaching procedure for the experimental groups (Fresh CP, Reapplied CP, Fresh TiO₂, and Reapplied TiO₂).

Group	pH Before Bleaching	pH After Bleaching
Fresh CP	6.0	7.0
Reapplied CP	6.0	7.0
Fresh TiO₂	6.0	7.0
Reapplied TiO₂	6.0	7.0

## Discussion

The fresh TiO₂ suspension exhibited a lower bleaching potential than the other groups; however, after reactivation, its efficacy increased markedly, suggesting that photocatalysis can enhance whitening outcomes. Both fresh and reapplied CP produced color changes comparable to those of reapplied TiO₂, indicating that TiO₂ reactivation effectively restores its bleaching potential. Therefore, the first hypothesis was rejected, as fresh TiO₂ did not exhibit color changes similar to those of fresh CP. In contrast, the second hypothesis was accepted, as the reuse of the suspension after UV light reactivation proved effective, with the reapplied TiO₂ group showing the highest color change values.

At-home bleaching with 16% CP remains a standard approach ^3^ ^,^ ^21^. CP decomposes into hydrogen peroxide (H₂O₂) and urea upon contact with water, saliva, or oral tissues, a process known as heterogeneous degradation ^3^ ^,^ ^21^. Urea further breaks down into ammonia and carbon dioxide, increasing the solution's pH ^3^ ^,^ ^21^ ^,^ ^22^. H₂O₂ primarily acts by oxidation, generating free radicals that diffuse into enamel and dentin, cleaving unsaturated chromophores into saturated molecules that scatter light more effectively, producing a whiter appearance ^3^ ^,^ ^21^ ^,^ ^23^.

Application of fresh CP for 2 hours daily over 21 days produced significant color change values, consistent with its mechanism of action. The 16% concentration was selected to balance bleaching efficacy and minimize sensitivity. Cardoso PC et al. (2010) reported that reducing application time from 8 to 1-2 hours daily decreases adverse effects without compromising results, attributed to the faster initial degradation of CP, 3.4 times higher in the first hour compared to subsequent periods. Once peroxide and oxygen saturate the tooth, the reaction slows, reducing the benefit of extended contact ^26^. This explains why the reused CP group showed efficacy similar to that of fresh CP, as unreacted peroxide can continue the whitening reaction even after initial use ^26^.

CP bleaching efficiency is pH-dependent, as higher pH accelerates peroxide decomposition and free radical release. In contrast, acidic conditions enhance peroxide stability but increase diffusion into dental tissues and the risk of sensitivity ^22^ ^,^ ^24^ ^,^ ^25^ ^,^ ^26^ ^,^ ^27^. In this study, a CP gel with a pH of six was used; its pH returned toward neutrality after application, and reapplication in contact with the tooth structure further favored radical release and sustained whitening. Upon contact with dentin, the pH increased to approximately 7.0, likely due to neutralization of H⁺ by hydroxyapatite and release of OH⁻ ions from the dentin matrix, illustrating a natural acid-base buffering effect that may influence CP activity.

Despite CP’s effectiveness, peroxide diffusion through dentinal tubules can elicit pulpal inflammation and sensitivity ^23^. Previous studies have shown that 16% CP can induce cytotoxic effects on pulp cells even after a single application ^30^ ^,^ ^31^. To minimize these effects, recent research has explored alternative agents such as TiO₂, nitrogen, and chitosan to enhance H₂O₂ performance without altering enamel morphology or inducing sensitivity ^32^ ^,^ ^33^ ^,^ ^34^. TiO₂, a semiconductor with valence and conduction bands, acts as a photocatalyst with oxidizing properties ^35^ ^,^ ^36^. When exposed to UV or blue-violet light (~388 nm), it excites electrons, generating oxygen ions and producing superoxide, resulting in a bleaching effect. TiO₂ is biocompatible, non-toxic, and does not alter pH as H₂O₂ does. In this study, the reapplied TiO₂ group exhibited the highest color change, likely due to enhanced radical generation and catalytic activity. TiO₂ particle size, morphology, and surface area influence photocatalytic performance ^17^ ^,^ ^37^ ^,^ ^38^, and the nanometric shape used here likely increased the surface-to-volume ratio, optimizing radical formation.

The photocatalytic process of TiO₂ involves UV absorption (λ ≤ 385 nm), promoting electron excitation from the valence to the conduction band ^15^ ^,^ ^16^ ^,^ ^39^ ^,^ ^40^ ^,^ ^41^ ^,^ ^42^. The resulting electron-hole pairs participate in redox reactions, producing hydroxyl radicals-potent oxidants with a redox potential of +2.8 V ^40^ ^,^ ^41^. The UV light used (385 nm) matched the peak absorption wavelength, ensuring effective excitation and radical generation.

The TiO₂ concentration (10%) was selected based on pilot data showing optimal results at this level and a 50-minute exposure duration. Extended exposure (100 minutes) further enhanced whitening, likely due to increased radical availability ^14^ ^,^ ^15^. Thus, activation of 10% TiO₂ for at least 100 minutes is recommended for bleaching applications. Future studies should evaluate different concentrations, UV wavelengths, and activation times to optimize efficiency.

According to the color variation results (Table 3), the fresh TiO₂ suspension exhibited significantly lower ΔE00 values than 16% carbamide peroxide (p < 0.05), indicating limited bleaching potential upon initial application. However, after reapplication and UV reactivation, TiO₂ showed a mean color change statistically comparable to that of the reapplied CP group, confirming that the photocatalytic mechanism becomes more efficient with extended UV exposure. These findings support the hypothesis that TiO₂ requires prior activation and cumulative energy input to reach its full whitening potential. The ΔE00 values obtained for the reapplied groups exceed the established perceptibility and acceptability thresholds, confirming that both treatments produced clinically perceptible and acceptable color changes. In contrast, the fresh TiO₂ group remained below these limits.

**Table 3:** Means (standard deviations) of color variation expressed in ΔE₀₀ (CIEDE2000) and pH.

Groups	PC16%	50%Tio_2_
Fresh	6.64 (0.51) A	2.36 (0.68) B
Reapplied	7.07 (0.50) A	7.67 (0.88) A

Different uppercase letters indicate statistically significant differences among groups within each parameter (p < 0.05).

From a clinical and commercial standpoint, the relatively long activation time (100 minutes) could be addressed through industrial pre-activation of the TiO₂ suspension, allowing the product to be commercialized in a ready-to-use form for direct dental application, without requiring additional light exposure by clinicians or patients. This would make the procedure more practical for both professional and at-home bleaching kits. TiO₂ nanoparticles are widely available from chemical suppliers and are low-cost, stable, and easy to incorporate into gel or syringe formulations. Therefore, industrial UV activation and packaging under controlled conditions could ensure product stability and performance, making TiO₂-based bleaching systems a feasible, safe, and sustainable alternative for clinical use ^35^ ^,^ ^36^ ^,^ ^37^ ^,^ ^38^ ^,^ ^40^ ^,^ ^41^ ^,^ ^42^.

Methodological limitations should also be acknowledged. This study used bovine teeth, which, although commonly employed as substitutes for human enamel, present microstructural and permeability differences that may influence bleaching outcomes. Furthermore, the absence of a negative control group and a UV-only group limits the ability to isolate the specific contribution of light exposure apart from TiO₂ photocatalysis. Wax was applied solely to fill the root canal space and serve as a sealing material during sample preparation. Since bleaching reactions occur primarily in enamel and dentin, and whitening agents diffuse through interprismatic and dentinal tubular pathways, the presence of wax in the root canal is unlikely to affect color change results. Finally, the lack of a detailed justification for the selected TiO₂ concentration (10%) may limit the generalizability of these findings, underscoring the need for future studies that explore a broader range of concentrations, UV wavelengths, and activation parameters.

In addition, using only one UV wavelength (385 nm) and two exposure times (50 and 100 minutes) limited the assessment of TiO₂'s full photocatalytic potential. Nonetheless, the results demonstrated that the TiO₂ suspension, even without hydrogen peroxide, promoted effective whitening when reactivated with UV light for 100 minutes. Therefore, TiO₂ emerges as a promising peroxide-free bleaching alternative for at-home use, warranting further longitudinal investigations and formulation optimization.

## Conclusion

Within the limitations of this study, it can be concluded that TiO₂ suspension exhibited effective bleaching potential only when reapplied and reactivated under UV light for a total of 100 minutes. Under these conditions, the color change achieved by the reapplied TiO₂ group was comparable to that observed with 16% carbamide peroxide. Therefore, TiO₂ demonstrates potential as a peroxide-free alternative for dental bleaching when appropriately activated by ultraviolet light. 

## Data Availability

The data supporting this study are available from the corresponding author upon reasonable request.
